# Oil content analysis of corn seeds using a hand-held Raman spectrometer and spectral peak decomposition algorithm

**DOI:** 10.3389/fpls.2023.1174747

**Published:** 2023-04-03

**Authors:** Yuan Jin, Hongwu Tian, Zhen Gao, Guiyan Yang, Daming Dong

**Affiliations:** ^1^ School of Computer, Electronics and Information, Guangxi University, Nanning, China; ^2^ Key Laboratory of Agricultural Sensors, Ministry of Agriculture and Rural Affairs, Beijing, China; ^3^ Research Center of Intelligent Equipment, Beijing Academy of Agriculture and Forestry Sciences, Beijing, China; ^4^ College of Information and Electrical Engineering, China Agricultural University, Beijing, China; ^5^ College of Plant Science and Technology, Huazhong Agricultural University, Wuhan, China

**Keywords:** Raman spectroscopy, spectral peak decomposition, Gaussian curve fitting, corn seed, oil content

## Abstract

Rapid, non-destructive and reliable detection of the oil content of corn seeds is important for development of high-oil corn. However, determination of the oil content is difficult using traditional methods for seed composition analysis. In this study, a hand-held Raman spectrometer was used with a spectral peak decomposition algorithm to determine the oil contents of corn seeds. Mature and waxy Zhengdan 958 corn seeds and mature Jingke 968 corn seeds were analyzed. Raman spectra were obtained in four regions of interest in the embryo of the seed. After analysis of the spectra, a characteristic spectral peak for the oil content was identified. A Gaussian curve fitting spectral peak decomposition algorithm was used to decompose the characteristic spectral peak of oil at 1657 cm^−1^. This peak was used to determine the Raman spectral peak intensity for the oil content in the embryo and differences in the oil contents among seeds of varying maturity and different varieties. This method is feasible and effective for detection of corn seed oil.

## 1 Introduction

Corn is a leading food crop in China. Corn kernel is mainly composed of seed coat, endosperm and embryo, which contains starch, oil, protein, cellulose, lignin, and soluble sugar (Chen, 2009). Corn is a major source of human food, animal feed, and industrial raw materials (Abbassian, 2006). Corn oil is extracted from the germ of the corn kernel and is rich in unsaturated fatty acids and vitamin E, which are essential in the human body (Aksoz et al., 2020; Zhao et al., 2020). This oil is a popular, high-quality edible oil. The oil content of common corn is approximately 4%, while the oil content of high-oil corn is greater than 8% (Barrera-Arellano et al., 2019). Therefore, increasing the oil content and quality in corn is crucial for improving its value. At the same time, determining the oil content in corn is important for selective breeding of high-oil corn and transgenic engineering (Barrera-Arellano et al., 2019). However, traditional analytical methods (Matthäus et al., 2001), such as solvent extraction, accelerated solvent extraction, supercritical fluid extraction, microwave-assisted extraction, and Soxtherm extraction, are destructive, time-consuming, labor-intensive and use many chemical reagents. These methods are not suitable for rapid and non-destructive quality evaluation of mass-produced corn seeds. Consequently, it is necessary to develop rapid and non-destructive techniques for quality evaluation of corn seeds.

Spectroscopy is a rapid and non-destructive detection method (Huang et al., 2015), and has been successfully applied to the quality evaluation of agricultural products. Among them, near infrared spectroscopy (NIR) is an absorption spectrum that has been used to rapidly determine the compositions of grain seeds, including the oil content (Fassio et al., 2015), total starch content of corn seeds (Liu et al., 2020), and protein content of cowpeas (Weng et al., 2017). However, NIR is mainly related to frequency doubling and combined vibrations of hydrogen-containing chemical groups (e.g., C-H, O-H, and N-H) in organic molecules (Beć et al., 2020). Consequently, this technique suffers from serious overlap of spectral peaks, low sensitivity, and is susceptible to interference from water. It is difficult to directly analyze the chemical compositions of seeds using the absorption peak characteristics of a NIR spectrum, and this has resulted in a dependence on chemometrics for analysis.

Raman spectroscopy is an analytical technique that is based on Raman scattering, which originates from the vibration and rotation of molecules (Jones et al., 2019).The positions, intensities and shapes of the spectral peaks can reflect the characteristic fundamental frequency vibrations of the functional groups or chemical bonds in molecule of the target substance. Compared with NIR, Raman spectroscopy has high sensitivity, produces clear and sharp spectral peaks, and provides strong recognition ability, which makes the analysis of chemical composition more intuitive and concise. Wu X. et al. (2022) established a quantitative model based on Raman spectra and one-dimensional convolutional neural network (1D CNN) to identify the amount of olive oil in a corn-olive oil blend, providing a new analytical method for the quantitative identification of vegetable oils. Yang et al. (2018) showed that characteristic peaks related to corn starch, an oil–starch mixture, zeaxanthin, lignin, and oil were located at 477, 1443, 1522, 1596, and 1654 cm^−1^, respectively. They realized rapid visual detection of the chemical composition of corn seeds using a line-scanning Raman hyperspectral imaging system. These studies indicate that Raman spectroscopy can be used to analyze the composition of corn seeds.

In recent years, because of continuous improvement in the performance of optical devices and other components, hand-held Raman spectrometers, which are small, flexible to use, simple to operate, and provide stable performance, have been favored by researchers. Farber and Kurouski (2018) analyzed the Raman spectra of corn seeds before and after pathogen infection using a hand-held Raman spectrometer. After infection, a peak at 1633 cm^–1^ for C=C vibration in the aromatic ring of lignin disappeared, which indicating that the lignin degraded. Furthermore, a peak at 1658 cm^–1^ belonging to the protein amide I band became stronger, which showed that growth of the pathogen was closely related to deposition of protein in corn. Additionally, spectral peaks related to the -C=C-plane vibration of carotenoid shifted and increased in intensity. Therefore, the growth of pathogens may be related to degradation and breaking of bonds in carotenoid. These studies show that a hand-held Raman spectrometer may be feasible for seed composition analysis. Although Raman spectroscopy can be used simultaneous measurement of various compositions of seeds, the Raman characteristic peaks of oil in corn seeds suffer from interference from starch, protein, lignin, and other compositions (Yang et al., 2018). To overcome this issue, Raman spectroscopy could be combined with a spectral peak decomposition algorithm to realize the identification, classification, and quantification of composition (Postnikov et al., 2021). Sadat and Joye (2020) uaed a peak decomposition method based on the second derivative of the original spectrum and the curve fitting of the Voigt function to identify, separate and quantify hidden peaks of the amide I band in the infrared and Raman spectra of globular proteins, hydrated zein and gluten proteins. Wu T. et al. (2022) proposed an optimal multi-peak fitting model for the first-order and second-order Raman spectra of high-strength carbon fibers (T-series) and high-strength and high-modulus carbon fibers (MJ series), and quantitatively analyzed the structure of the carbon fiber.

The primary aim of this study was to realize the oil content analysis of corn seeds using a hand-held Raman spectroscopy combined with a peak decomposition algorithm. The Raman spectral characteristics of the corn seed embryo were studied, and spectral information for the oil content in the corn seeds was extracted and analyzed using a spectral peak decomposition algorithm. Differences in the oil content for corn seeds of different varieties and maturities were compared and analyzed.

## 2 Materials and methods

### 2.1 Experimental materials

Corn seeds of the Zhengdan 958 and Jingke 968 varieties were obtained from a seed company in Beijing, China. Fifty seeds of the Zhengdan 958 variety were selected and divided into two groups according to the milk line of endosperm. 25 of the seeds were mature and the others were waxy. For the Jingke 968 variety, 25 mature seeds were selected. Standard samples, including corn starch (reagent grade, Aladdin Reagent Co., Ltd., Shanghai, China), corn oil (reagent grade, Aladdin Reagent Co., Ltd., Shanghai, China), cellulose (reagent grade, Aladdin Reagent Co., Ltd., Shanghai, China), and corn hulls, were obtained for analyzing the Raman characteristics of the corn seeds.

### 2.2 Instruments

Raman spectra of the standard samples were collected using a high-resolution benchtop Raman spectrometer (DXR Smart Raman System, Thermo Fisher Scientific). The spectrometer was equipped with a 780 nm diode laser with a maximum power of 150 mW. The spectral resolution was 3.0-4.1 cm^–1^, and the spectral range was 50-1800 cm^–1^. The optical power was 100 mW and the integration time was 10 s.

Raman spectra of the corn seeds were collected using a hand-held Raman spectrometer (785 hand-held Raman spectrometer, Beijing Yunduan Optical Technology Co., Ltd.). The hand-held Raman spectrometer was equipped with a 785 nm laser. The laser power was continuously adjustable from 1 to 500 mW. The spectral resolution was approximately 8.0 cm^–1^, and the spectral range is 200-1800 cm^–1^. The optical power was 150 mW and the integration time was 10 s.

### 2.3 Spectral data acquisition

**Figure 1:**
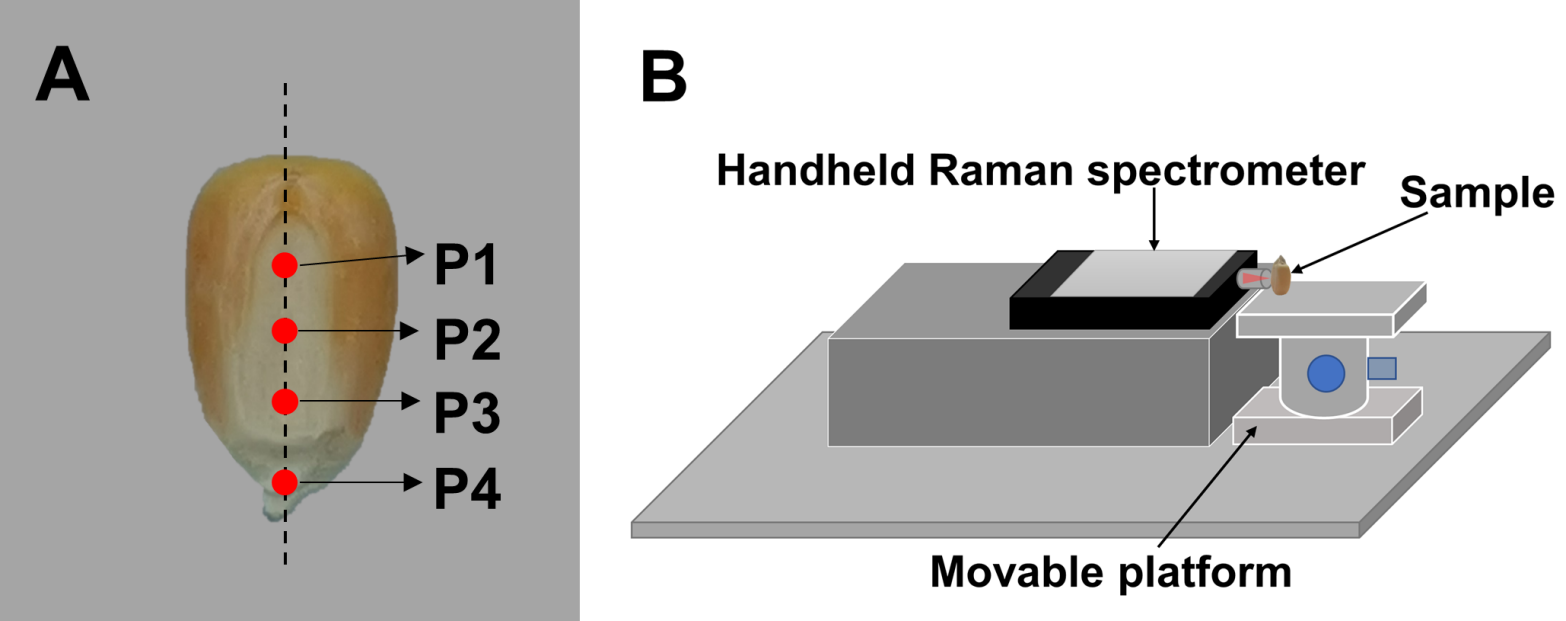
Schematic diagram of the Raman spectrum acquisition. **(A)** Locations of the four regions of interest in the embryo for detection, and **(B)** the acquisition system for the Raman spectra of the corn seeds.

Standard samples (corn starch, corn oil, cellulose, and hulls) were placed on a quartz plate, which was set on the sampling platform of DXR Smart Raman System for spectral acquisition. For the Zhengdan 958 seeds, the mature seeds were labeled as group D1 and the waxy seeds as group D2. The Jingke 968 seeds were labeled as group D3. Four regions of interest (ROIs) for detection (P1, P2, P3, and P4) were set from the top to bottom along the midline of the embryo of the corn seed (**Figure 1A**). The Raman spectra of the ROIs were collected by the acquisition system shown in **Figure 1B**. The acquisition system consisted of a hand-held Raman spectrometer, a movable platform, a sample rack, and an optical breadboard. The hand-held Raman spectrometer was placed horizontally on the left of the mobile platform. The seed sample was placed vertically on the right of the mobile platform, with the P1 end of the seed facing down and the tip of the embryo facing up. The embryonic surface of the seed faced the Raman probe. First, the laser was focused on P1 and Raman spectral data were collected. Then, the movable platform was adjusted to move the sample downward, and spectral data were collected at P2. This process was repeated for P3 and P4. During spectral collection, the four ROIs of each seed were as similar as possible. The spectra of the standard samples were used as reference data for the spectral analysis of the seeds.

### 2.4 Spectral data preprocessing

Firstly, the Savitzky–Golay smoothing algorithm was used to remove noise from the original Raman spectra of the corn seeds (Schafer, 2011). Then, the smooth Raman spectra were corrected using the airPLS algorithm to remove fluorescent background interference (Zhang et al., 2010). The corrected Raman spectral data were used for subsequent spectral peak decomposition and spectral information extraction. The preprocessing of all raw Raman spectra was performed in Visual Studio Code (Microsoft Corporation, Redmond, WA, United States).

### 2.5 Spectral peak decomposition

Decomposition of overlapping spectral peaks can be used for separation and extraction of effective spectral information. Previous studies have shown that curve fitting is an effective spectral peak decomposition method. It is based on statistical principles, which aims to find a reliable function to fit a set of data points and minimize the error between the function and the data point. And the least square method is a common functional form. In this study, curve fitting based on nonlinear least square method was selected for spectral peak decomposition, and the Gaussian linear function was selected as the fitting model. The expression of the Gaussian function is shown in Equation 1:


(1) $$\text{y}=\text{a}{\text{e}}^{-\frac{{\left(x-\mu \right)}^{2}}{2\sigma^{2}}}$$

where *a* is the peak intensity, *μ* is the peak position, and *σ* is the full width at half maximum. With three overlapping spectral peaks, the original spectral peaks were regarded as a ternary Gaussian linear distribution, and a function expression was constructed as shown in Equation 2:


(2) $$\text{y}={\text{a}}_{1}{\text{e}}^{-\frac{{\left(x-\mu_{1}\right)}^{2}}{2\sigma_{1}^{2}}}+{\text{a}}_{2}{\text{e}}^{-\frac{{\left(x-\mu_{2}\right)}^{2}}{2\sigma_{2}^{2}}}+{\text{a}}_{3}{\text{e}}^{-\frac{{\left(x-\mu_{3}\right)}^{2}}{2\sigma_{3}^{2}}}$$

According to Equation 2, the original overlapping spectral peaks were iteratively analyzed by curve fitting to obtain the values of the Gaussian parameters *a*_1_, *μ*_1_, *σ*_1_, *a*_2_, *μ*_2_, *σ*_2_, *a*_3_, *μ*_3_, and *σ*_3_. Then, three Gaussian spectral peaks $a_{1}e-\frac{{\left(x-\mu_{1}\right)}^{2}}{2\sigma_{1}^{2}},a_{2}e-\frac{{\left(x-\mu_{2}\right)}^{2}}{2\sigma_{2}^{2}},a_{3}e-\frac{{\left(x-\mu_{3}\right)}^{2}}{2\sigma_{3}^{2}}$ were obtained by decomposition of the overlapping peaks and used for information extraction. All overlapping peaks of Raman spectra were decomposed in Visual Studio Code.

## 3 Results and discussion

### 3.1 Analysis of Raman spectral characteristics of oil in corn seeds

**Figure 2:**
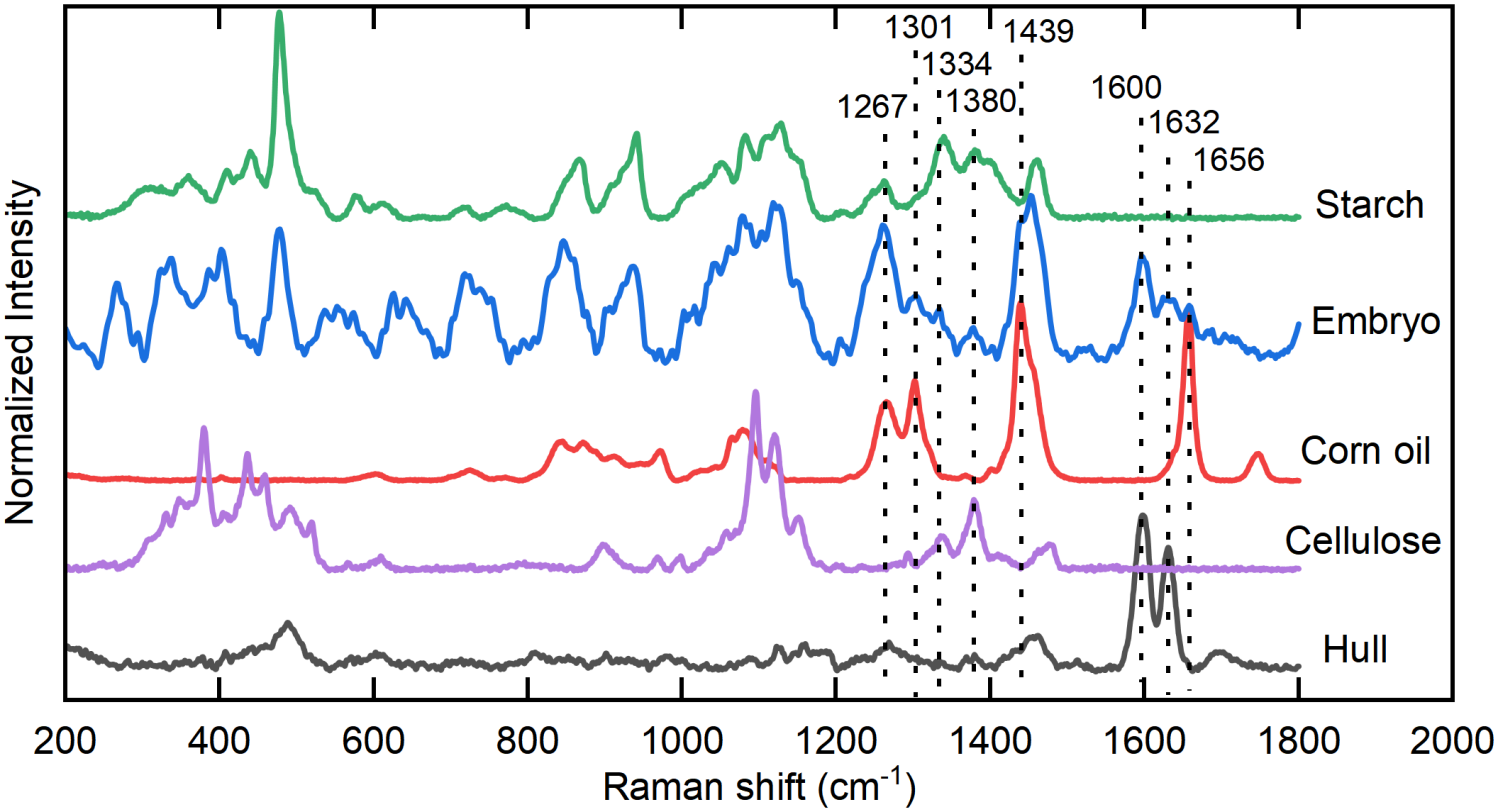
Analysis of the Raman spectral characteristics of oil in corn seeds. Characteristic peaks of corn embryo located at 1267, 1301, 1439, 1656 cm^–1^ are related to standard corn oil sample, 1334, 1380 cm^–1^ are related to standard cellulose sample, and 1600, 1632 cm^–1^ are related to hull. At the same time, 1267, 1301, 1334, 1380, 1439 cm^–1^ are affected by the peaks of starch.

Corn oil is mainly stored in the embryo of the seed. To analyze the Raman spectral characteristics of oil in the corn seeds, the standard spectra of corn oil, corn starch, cellulose, and hulls collected by the DXR Smart Raman system were regarded as the reference spectra and compared with the Raman spectra of the embryo collected by the hand-held Raman (**Figure 2**). Prominent characteristic peaks in the standard corn oil sample were located at 1656, 1439, 1301, and 1267 cm^–1^, which are attributed to C=C stretching, CH_2_ or CH_3_ deformation vibrations, CH_2_ twisting, and =C-H bending, respectively (Gelder et al., 2007; Anna et al., 2017). The 1656 cm^–1^ spectral peak in the seed embryo spectrum overlapped with spectral peaks at 1600 and 1632 cm^–1^, which were attributed to C-H stretching of the aromatic ring and C=C stretching in coniferyl aldehyde, respectively, derived from lignin in the hull (Lupoi and Smith, 2012; Zeng et al., 2016). In the spectrum of the seed embryo, there was serious overlapping between the 1439 cm^–1^ peak of corn oil and the band located at approximately 1460 cm^–1^ for CH_2_ bending of starch (Liu et al., 2004). Furthermore, the spectral peaks of corn oil located at 1301 and 1267 cm^–1^ overlapped with a peak at 1263 cm^–1^ related to starch (Kizil et al., 2002) and peaks at 1336 and 1379 cm^–1^ related to cellulose (Wiley and Atalla, 1987; Kryeziu et al., 2022). Compared with the standard spectrum of corn oil, the spectrum of the seed embryo had a very different ratio between the peaks at 1301 and 1267 cm^–1^. These results indicate that the 1656 cm^–1^ peak is the most suitable among the characteristic peaks for the spectral analysis of oil in corn seeds.

### 3.2 Raman peak decomposition analysis of oil in corn seeds

**Figure 3:**
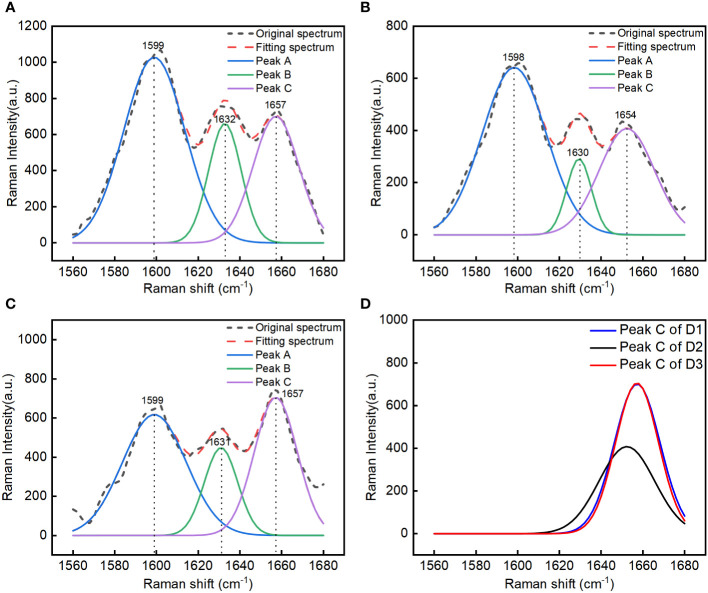
Spectral peak decomposition analysis of different kinds of corn seeds in the 1560-1680 cm^–1^ region: group D1 (mature Zhengdan 958) seeds **(A)**, group D2 (waxy Zhengdan 958) seeds **(B)**, and group D3 (Jingke 968) seeds **(C)**. Comparative analysis of the separated spectral peak for oil in the three groups of seeds **(D)**.

A Raman spectrum of the P1 ROI was selected from each group (D1, D2, and D3) of corn seeds, and the 1560–1680 cm^–1^ region was selected for spectral peak decomposition analysis to extraction information about the oil content. There were multiple overlapping peaks in the 1560–1680 cm^–1^ region (**Figure 2**). The original spectral peaks were regarded as a distribution of three Gaussian spectral peaks. A function expression was constructed as shown in Equation 2, and decomposition of the spectral peaks was carried out. The fitting parameters *a*_1_, *μ*_1_, *σ*_1_, *a*_2_, *μ*_2_, *σ*_2_, *a*_3_, *μ*_3_, and *σ*_3_ for the three Gaussian spectral peaks (A, B, and C) were calculated (**Table 1**). The decomposition results for the overlapping peaks are shown in **Figures 3A–C**. There were slight differences in the positions of the peaks among the three groups of the seeds. Peaks A and B were consistent with lignin, and peak C could be used to determine the oil content. Our results showed that the overlapping spectral peaks were successfully decomposed into three Gaussian spectral peaks for each group of corn seeds. The relative errors between the fitted and original spectra were 8.68%, 7.71%, and 12.72% for D1, D2, and D3, respectively. The relative error was obtained by subtracting the fitting value from the original value, dividing by the original value, then taking the absolute value of the result, adding all the absolute values, and dividing by the total number of data points. The Raman peak for the oil content was successfully separated from any overlapping spectral peaks using the peak decomposition algorithm (**Figure 3D**). The spectra of the mature seeds (groups D1 and D3) were similar, whereas that of the waxy seeds (group D2) was very different.

**Table 1:** Fitting parameters for spectral peak decomposition in the 1560-1680 cm^–1^ region for corn seed spectra.

Seed	Fitting parameters of spectral peak									Decomposition spectral peaks			Relative error
	*a*_1_	*μ*_1_	*_σ_*_1_	*a*_2_	*μ*_2_	*_σ_*_2_	*a*_3_	*μ*_3_	*_σ_*_3_	Peak A	Peak B	Peak C	
D1	1028	1599	15	661	1632	8	702	1657	10	$1028\times e^{-\frac{{\left(x-1599\right)}^{2}}{450}}$	$661\times e^{-\frac{{\left(x-1632\right)}^{2}}{128}}$	$702\times e^{-\frac{{\left(x-1657\right)}^{2}}{200}}$	8.68%
D2	642	1598	15	292	1630	6	408	1654	13	$642\times e^{-\frac{{\left(x-1598\right)}^{2}}{450}}$	$292\times e^{-\frac{{\left(x-1630\right)}^{2}}{72}}$	$408\times e^{-\frac{{\left(x-1654\right)}^{2}}{338}}$	7.71%
D3	618	1599	15	449	1631	8	705	1657	10	$618\times e^{-\frac{{\left(x-1599\right)}^{2}}{450}}$	$449\times e^{-\frac{{\left(x-1631\right)}^{2}}{128}}$	$705\times e^{-\frac{{\left(x-1657\right)}^{2}}{200}}$	12.72%

### 3.3 Analysis of the oil content in the embryos of different corn seeds

**Figure 4:**
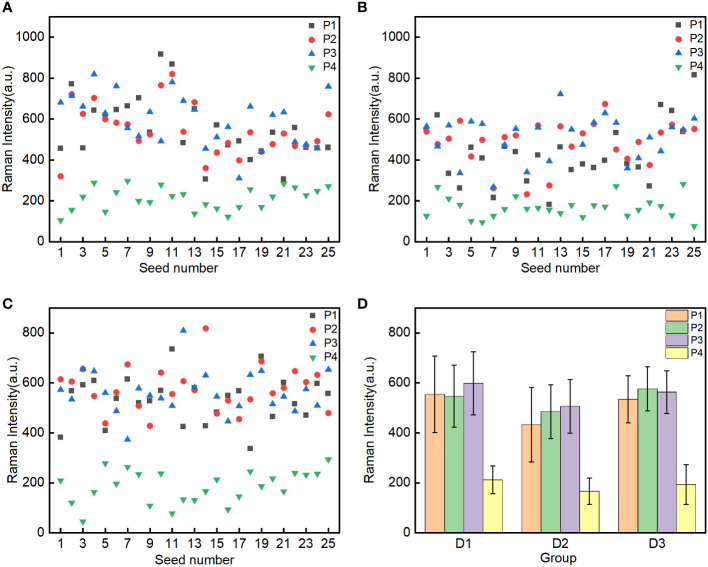
Spectral intensity analysis of peak C in the four regions of interest (P1-Point 1, P2-Point 2, P3-Point 3 and P4-Point 4 of embryo) in the three groups of seeds: mature Zhengdan 958 D1 **(A)**, waxy Zhengdan 958 D2 **(B)** and Jingke 968 D3 **(C)**. The mean spectral intensities in the P1, P2, P3, and P4 regions of interest in the embryo of three groups seeds **(D)**.

To analyze the Raman characteristics of the oil content in the embryos of different corn seeds, the Raman spectra of the different groups (D1, D2, and D3; 25 seeds for each group) were decomposed using the Gaussian curve fitting algorithm in the 1560–1680 cm^–1^ region. The maximum intensity of the decomposed peak C for oil was extracted for analysis. To analyze the different ROIs (P1, P2, P3, and P4) in the embryos, scatter plots of the maximum intensities of peak C were constructed (**Figures 4A–C**). The maximum intensities of peak C in the P1, P2, and P3 ROIs were scattered throughout the same region of the plots and these ROIs could not be clearly distinguished. By contrast, all data points for the P4 ROI were located at the bottom of the scatter plots and clearly separated from the data points for the P1, P2, and P3 ROIs.

To analyze the differences in the Raman intensities in the four ROIs, the mean intensities of peak C for the four ROIs for the 25 seeds in each group were calculated. The distribution of mean intensities and the corresponding standard deviations for the three groups of seeds are shown in **Figure 4D**. There were slight differences in the mean spectral intensities of peak C for the P1, P2, and P3 ROIs in the embryos of the three groups of corn seeds. However, the mean intensity of peak C for the P4 ROI was significantly lower than in the P1, P2, and P3 ROIs. This difference is consistent with the fact that corn oil is mainly distributed in the germ (Moreau and Hicks, 2005) and shows that the Raman peak at 1657 cm^–1^ can be used to characterize the oil content. To better characterize the oil content in the seeds and improve the stability and reliability of detection, the mean maximum intensity of the 1657 cm^–1^ peak in the P1, P2 and P3 ROIs was selected.

**Figure 5:**
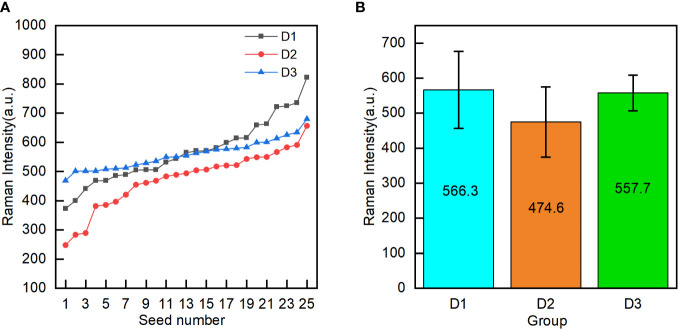
Oil content characterization values (the mean intensity of peak C in the embryo) of individual seeds in the three groups (D1-mature Zhengdan 958, D2-waxy Zhengdan 958, and D3-Jingke 968) **(A)**, and the mean oil content characterization value for each group **(B)**.

To analyze the difference in the oil content between the groups of corn seeds, the oil characterization value was calculated for every individual seed in each group. The characterization values of the 25 seeds in each group were arranged in ascending order (**Figure 5A**). The characteristic values of the seeds in groups D1 and D2 fluctuate greatly with the number of seeds, while those of the D3 seeds are relatively stable. The characteristic values of the seeds in groups D1 and D2 were spread over a wider range than those for the D3 seeds. These results showed that the oil content of individual seed in the D1 and D2 groups is significantly different, and the oil content in the D3 group has little difference among individual seeds. To analyze the differences in the oil contents among the three groups of corn seeds, the mean oil characterization values of all 25 seeds in each group were calculated to characterize the overall oil content of each group (**Figure 5B**). The seeds in group D2 had the lowest mean value (474.6). The seeds in group D1 had a mean value of 566.3, which was significantly higher than that of the seeds in group D2 and not significantly different from that of the seeds in group D3 (557.7). The seeds in group D1 and group D2 were the same variety (Zhengdan 958). For seeds of the same variety, the relative oil content was correlated with the seed maturity, with the oil content of mature seeds being higher than that of waxy seeds. Among different varieties, the oil content in the Jingke 968 (D3 group) was similar to that in the Zhengdan 958 variety; the results for the Jingke 968 variety showed less variation among the individual seeds in the group than was observed for the Zhengdan 958 variety.

## 4 Conclusions

The feasibility of detecting the oil content of corn seeds using a hand-held Raman spectrometer with a spectral peak decomposition algorithm was explored. The Raman peak at 1657 cm^–1^ for the oil content was successfully separated from overlapping spectral peaks using a Gaussian curve fitting peak decomposition algorithm. The intensity distribution characteristics of the 1657 cm^–1^ spectral peak in different ROIs of the embryo showed that the oil content of the corn seed was mainly distributed in the germ. The oil content for individual seeds was characterized using the maximum intensity of the 1657 cm^–1^ peak. For the same variety of corn seeds, the oil content was positively correlated with seed maturity, with the oil content of mature seeds being higher than that of waxy seeds. For different varieties of corn seeds, the oil content of Jingke 968 variety was similar to that of Zhengdan 958 variety, but the oil contents of individual seeds of the Jingke 968 variety showed less variation within the group than was observed for the Zhengdan 958 variety. Our results show that a hand-held Raman spectrometer combined with spectral peak decomposition can provide rapid and non-destructive determination of the oil content of corn seeds. This study provides a basis for the quantitative detection of oil in corn or other varieties of grain. This method could be used to rapidly identify and select corn seeds with high oil contents for selective breeding.

## Data availability statement

The original contributions presented in the study are included in the article/supplementary material. Further inquiries can be directed to the corresponding author.

## Author contributions

YJ, HT, ZG, GY and DD contributed to the conception and design of the study. YJ and HT carried out the experiment and analyzed the experimental data. YJ and ZG wrote the first draft of the manuscript. GY and DD presented the revision of the manuscript. All authors contributed to the article and approved the submitted version.

## References

[B1] Abbassian A. (2006). Maize International Market Profile (Rome: Food and Agriculture Organization of the United Nations), 1–37.

[B2] Aksoz E. Korkut O. Aksit D. Gokbulut C. (2020). Vitamin e (α-, β + γ- and δ-tocopherol) levels in plant oils. Flavour Frag. J. 35 (5), 504–510. doi: 10.1002/ffj.3585

[B3] Anna I. Bartosz P. Lech P. Halina A. (2017). Novel strategies of raman imaging for brain tumor research. Oncotarget 8 (49), 85290. doi: 10.18632/oncotarget.19668 29156720 PMC5689610

[B4] Barrera-Arellano D. Badan-Ribeiro A. P. Serna-Saldivar S. O. (2019). “Corn oil: composition, processing, and utilization,” in Corn, ed. Serna-Saldivar S. O. (Amsterdam: AACC International Press), 593–613. doi: 10.1016/B978-0-12-811971-6.00021-8

[B5] Beć K. B. Grabska J. Huck C. W. (2020). NIR spectroscopy of natural medicines supported by novel instrumentation and methods for data analysis and interpretation. J. Pharm. Biomed. Anal. 193, 113686. doi: 10.1016/j.jpba.2020.113686 33142115

[B6] Chen J. (2009). Maize starch industry Manual (Fine). (Beijing: China Light Industry Press).

[B7] Farber C. Kurouski D. (2018). Detection and identification of plant pathogens on maize kernels with a hand-held raman spectrometer. Anal. Chem. 90 (5), 3009–3012. doi: 10.1021/acs.analchem.8b00222 29461798

[B8] Fassio A. S. Restaino E. A. Cozzolino D. (2015). Determination of oil content in whole corn ( zea mays l.) seeds by means of near infrared reflectance spectroscopy. Comput. Electron. Agr. 110, 171–175. doi: 10.1016/j.compag.2014.11.015

[B9] Gelder J. D. Gussem K. D. Vandenabeele P. Moens L. (2007). Reference database of raman spectra of biological molecules. J. Raman Spectrosc. 38 (9), 1133–1147. doi: 10.1002/jrs.1734

[B10] Huang M. Wang Q. G. Zhu Q. B. Qin J. W. Huang G. (2015). Review of seed quality and safety tests using optical sensing technologies. Seed Sci. Technol. 43 (3), 337–366. doi: 10.15258/sst.2015.43.3.16

[B11] Jones R. R. Hooper D. C. Zhang L. Wolverson D. Valev. V. K. (2019). Raman techniques: Fundamentals and frontiers. Nanoscale Res. Lett. 14 (1), 1–34. doi: 10.1186/s11671-019-3039-2 31300945 PMC6626094

[B12] Kizil R. Irudayaraj J. Seetharaman K. (2002). Characterization of irradiated starches by using FT-raman and FTIR spectroscopy. J. Agric. Food Chem. 50 (14), 3912–3918. doi: 10.1021/jf011652p 12083858

[B13] Kryeziu A. Slovak V. Parmentier J. Zelenka T. Rigolet S. (2022). Porous carbon monoliths from ice-NaOH templated dissolved cellulose. Ind. Crop Prod. 183, 114961. doi: 10.1016/j.indcrop.2022.114961

[B14] Liu Y. Himmelsbach D. S. Barton F. E. (2004). Two-dimensional Fourier transform raman correlation spectroscopy determination of the glycosidic linkages in amylose and amylopectin. Appl. Spectrosc. 58 (6), 745–749. doi: 10.1366/000370204873006 15198829

[B15] Liu C. Huang W. Yang G. Wang Q. Li J. (2020). Determination of starch content in single kernel using near-infrared hyperspectral images from two sides of corn seeds. Infrared Phys. Techn. 110, 103462. doi: 10.1016/j.infrared.2020.103462

[B16] Lupoi J. S. Smith E. A. (2012). Characterization of woody and herbaceous biomasses lignin composition with 1064 nm dispersive multichannel raman spectroscopy. Appl. Spectrosc. 66 (8), 903–910. doi: 10.1366/12-06621 22800567

[B17] Moreau R. A. Hicks K. B. (2005). The composition of corn oil obtained by the alcohol extraction of ground corn. J. Am. Oil Chem. Soc. 82 (11), 809–815. doi: 10.1007/s11746-005-1148-4

[B18] Postnikov E. B. Lebedeva E. A. Zyubin A. Y. Lavrova A. I. (2021). The cascade Hilbert-zero decomposition: A novel method for peaks resolution and its application to raman spectra. Mathematics 9 (21), 2802. doi: 10.3390/math9212802

[B19] Sadat A. Joye I. J. (2020). Peak fitting applied to Fourier transform infrared and raman spectroscopic analysis of proteins. Appl. Sci. 10 (17), 5918. doi: 10.3390/app10175918

[B20] Schafer R. W. (2011). What is a savitzky-golay filter? [Lecture notes]. IEEE Signal Proc. Mag. 28 (4), 111–117. doi: 10.1109/MSP.2011.941097

[B21] Matthäus B. Brühl L. (2001). Comparison of different methods for the determination of the oil content in oilseeds. J. Am. Oil Chem. Soc. 78 (1), 95–102. doi: 10.1007/s11746-001-0226-y

[B22] Weng Y. Shi A. Ravelombola W. S. Yang W. Qin J. Motes D.. (2017). A rapid method for measuring seed protein content in cowpea ( vigna unguiculata (L.) walp). Am. J. Plant Sci. 8 (10), 2387. doi: 10.4236/ajps.2017.810161

[B23] Wiley J. H. Atalla R. H. (1987). Band assignments in the raman spectra of celluloses. Carbohyd. Res. 160, 113–129. doi: 10.1016/0008-6215(87)80306-3

[B24] Wu X. Gao S. Niu Y. Zhao Z. Ma R. Xu B.. (2022). Quantitative analysis of blended corn-olive oil based on raman spectroscopy and one-dimensional convolutional neural network. Food Chem. 385, 132655. doi: 10.1016/j.foodchem.2022.132655 35279503

[B25] Wu T. Lu C. Sun T. Li Y. (2022). Study on raman multi-peak fitting and structure quantitative analysis of PAN-based carbon fibers. J. Mater. Sci. 57 (32), 15385–15412. doi: 10.1007/s10853-022-07589-8

[B26] Yang G. Wang Q. Liu C. Wang X. Fan S. Huang W. (2018). Rapid and visual detection of the main chemical compositions in maize seeds based on raman hyperspectral imaging. Spectrochim. Acta A. 200, 186–194. doi: 10.1016/j.saa.2018.04.026 29680497

[B27] Zeng Y. Yarbrough J. M. Mittal A. Tucker M. P. Vinzant T. B. Decker S.. (2016). *In situ* label-free imaging of hemicellulose in plant cell walls using stimulated raman scattering microscopy. Biotechnol. Biofuels 9 (1), 1–16. doi: 10.1186/s13068-016-0669-9 27895710 PMC5120481

[B28] Zhang Z. M. Chen S. Liang Y. Z. (2010). Baseline correction using adaptive iteratively reweighted penalized least squares. Analyst 135 (5), 1138–1146. doi: 10.1039/b922045c 20419267

[B29] Zhao M. Lan Y. Cui L. Monono E. Rao J. Chen B. (2020). Formation, characterization, and potential food application of rice bran wax oleogels: Expeller-pressed corn germ oil versus refined corn oil. Food Chem. 309, 125704. doi: 10.1016/j.foodchem.2019.125704 31699556

